# F-Ratio Test and Hypothesis Weighting: A Methodology to Optimize Feature Vector Size

**DOI:** 10.1155/2011/290617

**Published:** 2011-08-17

**Authors:** R. M. Dünki, M. Dressel

**Affiliations:** ^1^Physics Institute, CAP, University of Zürich, CH 8057 Zürich, Switzerland; ^2^Research Department, Cantonal Psychiatric Hospital, CH 8462 Rheinau, Switzerland; ^3^Department of Psychology, University of Konstanz, 78457 Konstanz, Germany; ^4^Verhaltenstherapie, Post-Straße 3, 79098 Freiburg, Germany

## Abstract

Reducing a feature vector to an optimized dimensionality is a common problem in biomedical signal analysis. This analysis retrieves the characteristics of the time series and its associated measures with an adequate methodology followed by an appropriate statistical assessment of these measures (e.g., spectral power or fractal dimension). As a step towards such a statistical assessment, we present a data resampling approach. The techniques allow estimating *σ* ^2^(*F*), that is, the variance of an *F*-value from variance analysis. Three test statistics are derived from the so-called *F*-ratio *σ* ^2^(*F*)/*F* ^2^. A Bayesian formalism assigns weights to hypotheses and their corresponding measures considered (hypothesis weighting). This leads to complete, partial, or noninclusion of these measures into an optimized feature vector. We thus distinguished the EEG of healthy probands from the EEG of patients diagnosed as schizophrenic. A reliable discriminance performance of 81% based on Taken's *χ*, *α*-, and *δ*-power was found.

## 1. Introduction 

The reduction of a feature vector to an optimized dimensionality is a common problem in the context of signal analysis. Consider for example, the assessment of the dynamics of biomedical/biophysical signals (e.g., EEG time series). These may be assessed with either linear (mainly: power spectral) and/or nonlinear (mainly: fractal dimension) analysis methods [1–5]. Each of the methods used for analysis of the time series extracts one or several measures out of a signal like peak frequency, band power, correlation dimension, K-entropy, and so forth. Some, but not necessarily all of these measures are supposed to exhibit state-specific information connected to the underlying biological/physiological process. Let us denote a collection of these measures a feature vector. An appropriately weighted collection of these information, specific measures may span an optimal feature vector in the sense that the states may be best separated. 

The temporal variation of these signals often has to be regarded as being almost stationary over limited segments only and not as being stationary in a strict sense, a property which is sometimes denoted as “*quasistationarity*”. This suggests regarding a specific outcome as being randomly drawn from a distribution of outcomes around a state-specific mean. Hence any inference made on such outcomes must be based on statistics relating the effect of interest to that stochastic variation even when regarding a single individual. If a comparative study is conducted, one has to select samples of probands, and this again introduces sources of random variations into analysis. The problem to solve is hence twofold. Efforts must be made (1) to retrieve effects out of the random variations for the different measures and (2) to reduce the set of all measures to the set of those which allow for a reliable state identification. 

A widespread statistical method used to attack the first type of problem is known as analysis of variance. Given the *i*th measurement of a biophysical/biomedical signal, the perhaps most simple variance analytic model for this signal reads as

(1) $$\begin{array}{c} \text{signa}{\text{l}}_{ji}=\alpha_{j}+\text{erro}{\text{r}}_{i} \end{array},$$

where *i* denotes the *i*th measurement of the signal which was obtained under experimental condition *j*. The so-called effect (or treatment) term *α*_*j*_ may be a fixed or a random effect and either continuous or discrete (cf. below). With regard to model (1), the analysis of variance infers the extent to which the estimates of the squared differences among the effects *α*_*j*_ rise above the squared error. Testing the significance of the effect then depends upon whether the levels *α*_*j*_ are regarded as fixed or random, whereby the null hypothesis is normally formulated as having equal levels.

A typical situation for this problem is when a study is based on a sample of probands. The probands must be viewed as a random sample drawn out of the reservoir of all possible individuals. 

If no correction is made, the analysis result applies specifically to the sample at the end. This is in most cases not the effect hunted for because one searches results applicable also to those (normally vast majority of) humans who were not included in the study, for example, reliable discriminant functions. The classical approach in variance analysis splits the effect term into two parts, fixed and random, and also enriches the error term with an estimate of the random part.

As an alternative to this classical approach, one may consider the family of the so-called *F-*ratio tests which are based on randomly splitting and recollecting the sample. One hereby chooses repeatedly random subsets of the original data to gain an estimate of the variance of *F*, namely, *σ* ^2^(*F*), and inspects the ratios *σ* ^2^(*F*)/*F* ^2^ or variants therefrom [6]. Here *F* denotes the quantity obtained from a *F-*test (cf.  Section 2.1). Such resampling methods have proven capabilities to enhance statistical inference on parameter estimates which are not available otherwise. The most popular examples of such methods are known as Jackknife or Bootstrap. *F-*ratio test statistics have indicated to (a) better retrieve fixed effects by fading away the random parts and (b) allow for an incremental test, that is, testing the effect of the inclusion of additional variables into an existing feature vector. The latter property makes them especially interesting when one tries to reduce the dimension of a feature vector to an optimal size. The different combinations with additional variables included lead to different probabilities under the hypotheses of interest which, in turn, allow for a weighted inclusion of these measures into an optimal feature vector. One may thus perform an adaptive model selection. 

A traditional way of model selection would be to perform analysis on all combination of features under interest and then to make a decision with the help of some information criterion (AIC, BIC, etc.). These try to select the optimal combination by weighting the number of measures in the model against residual error. This kind of selection leads to an inclusion of a measure with weight of either one or zero, however, and may neglect knowledge gained from incremental tests as those mentioned above. This pecularity motivated us to search for alternatives. Weighting information of different sources to an optimal degree is frequently conducted via Bayes' theorem. The Bayesian view will be adapted to derive weights different from zero and one for the construction of feature vectors, that is, to allow for partial inclusion. We note that reduced inclusion is also an important property of the so-called shrinkage or penalized regression methods [7].

The rest of the paper is organized as follows. We first recapitulate the derivation of three different *F-*ratio test statistics and outline the computational scheme to construct the corresponding confidence intervals by means of Monte Carlo simulations. A comparison to the outcome of the traditional method is made. We then show the inclusion of the outcome of these multivariate statistical methods into a selection scheme following a Bayesian heuristic by weighting hypotheses. This allows for reliably constructing weights for the measures. These weights are the basis for constructing reliable feature vectors suitable for further analysis, for example, discriminance procedures.

We demonstrate our approach on the reanalysis of an earlier study and address the problem of state specificity: psychosis versus nonpsychosis as expressed in the EEG. It is shown that an optimal combination of the so-called relative unfolding (or Taken's) *χ* and two power spectral estimates (*α*, *δ*) will allow for a correct classification of at least 81% of the probands, even in absence of active mental tasks.

## 2. Recapitulation of the *F*-Ratio Test

### 2.1. Recapitulation of ANOVA/MANOVA

The usage of analysis of variance is the traditional approach to distinguish systematic effects from noise. The methods of analysis of variance (ANOVA/MANOVA) try to decompose the variance of a population of outcomes (e.g., the results of EEG assessments obtained under different well-defined conditions) into two parts, namely, the treatment effect and the error effect. We adopt the notation of Bortz [8] and denote the treatment effect as *h* ^2^ and the error effect as *e* ^2^. The treatment effect *h* ^2^ explains how much of the total sum of squares may be due to a systematic effect of the different conditions (treatments). The second part, *e* ^2^, is an estimator of the remaining sum of squares due to other random or noise effects. In the light of (1), the term “error” affects both, *e* ^2^ and *h* ^2^, whereas *α* affects *h* ^2^ only [8]. The important question is: to what extent the treatment effect significantly rises above the level of a possible error effect. The quantity entering this test is (univariate case)

(2) $$\begin{array}{c} c=\frac{h^{2}}{e^{2}}. \end{array}$$

As stated above, *h* ^2^ denotes the sum of squares due to treatment and *e* ^2^ the sum of squares due to error. If the influence of the treatment is zero, *h* ^2^ also reflects only the error influence. Hence the test may be formulated as an *F-*test, that is, to test whether a calculated value of *F* might have occurred by chance or if the value deviates significantly from an outcome by chance. This might be done classically by comparing the evaluated value of *F* with the values in a table displaying *F-*value probabilities or get it from an appropriate statistical software package. 

The *F-*value is given as

(3) $$\begin{array}{c} F=\frac{cg}{g}\cdot \frac{df_{e}}{df_{h}}=\frac{h^{2}}{e^{2}}\cdot \frac{df_{e}}{df_{h}}:=\frac{\sigma_{h}^{2}}{\sigma_{e}^{2}} \end{array},$$

where *g* is some appropriate weight (without having an effect in the univariate case, however), and *df*_*e*_  and  *df*_*h*_ are the corresponding degrees of freedom, respectively. The univariate case (ANOVA) tests the influence of one or more treatment effects upon the outcome of a single variable, for example, how the nonlinear correlation-dimension estimate b_0_ [9] is affected by group, mental situation, and proband (cf.  Section 4).

The possible existence of an overall effect must be tested not only on *b*_0_ but also simultaneously on all evaluated measures, however. So the appropriate test is not a sequence of ANOVA tests but a multivariate approach (MANOVA). This is because the outcome of the variables might be statistically dependent to some degree, and thus the simultaneous effect is different from the set of the effects of the individual variables. Hence, (3) must be converted to the multivariate case. The quantities *h* ^2^ and *e* ^2^ turn into their corresponding matrices H and E [8]. The *F-test* depends now on the eigenvalues of the matrix HE^−1^ which is analogous to (3), but the single weight *g* splits up into the weights *g*_*i*_, and these may be different for different axes_*i*_. The most common of such *F-*values are

(4) $$\begin{array}{c} F_{\text{H}}=\frac{\sum_{i}^{s}c_{i}/\left(g\right)}{\sum_{i}^{s}1/\left(g\right)}\cdot \frac{df_{e}}{df_{h}} \end{array}$$

(i.e.,  *g*_*i*_ = 1/*g*  ∀*i*),

(5) $$\begin{array}{c} F_{P}=\frac{\sum_{i}^{s}c_{i}/\left(1+c_{i}\right)}{s-\sum_{i}^{s}c_{i}/\left(1+c_{i}\right)}\cdot \frac{df_{e}}{df_{h}} \end{array}$$

(i.e.,  *g*_*i*_ = 1/(1 + *c*_*i*_)), or

(6) $$\begin{array}{c} F_{R}=\frac{c_{1}/\left(1+c_{1}\right)}{1-c_{1}/\left(1+c_{1}\right)}\cdot \frac{df_{e}}{df_{h}} \end{array}$$

(i.e., *g*_1_ = 1/(1 + *c*_1_);  *g*_*i*_ = 0  ∀*i* ≥ 2), where *c*_*i*_ is the *i*th (ordered by value) eigenvalue of the matrix HE^−1^, and *s *= rank(HE^−1^). Equation (4) is known as Hotelling's (generalized) *T* ^2^, [10], (5) as Pillais' trace [11], and (6) as Roy's largest root [12]. For a sufficiently large number of observations, *F*_H_, *F*_*R*_, and *F*_*P*_ become equivalent and, in the *s *= 1 case, they become identical. As in the univariate case, testing for significance of an effect is done by evaluating the probability that a calculated *F-*value might occur by chance. The software packages that perform MANOVA do normally return this probability together with further properties on the sum of squares involved in H and E. 

### 2.2. Outline of the Problem Separating Fixed and Random Effects 

To motivate the derivation of our algorithm, we consider the influence of a randomly chosen sample of persons out of a population, whereby other effects might also be present, but fixed. The effect term *h* ^2^ may then be decomposed into

(7) $$\begin{array}{c} h^{2}={\left(\Delta a\right)}^{2}+{\left(\Delta pa\right)}^{2}+{\left(\Delta e\right)}^{2} \end{array},$$

where (Δ*a*)^2^ denotes here the influence of fixed conditions, (Δ*pa*)^2^ the effect of the (randomly chosen) persons, and (Δ*e*)^2^ the influence of the random error effects [8]. (We note that the quantities (Δ*a*)^2^ and (Δ*pa*)^2^ are sometimes also called treatment effects in a biomedical context). Under the null hypothesis of having no fixed effect, (Δ*a*)^2^ is assumed to be zero. The same holds—in principle—for (Δ*pa*)^2^. Generally, if an observable stems from a subpopulation drawn from a larger set, the corresponding effect may itself become random. This is normally the case when regarding person as condition (one will never be able to assess all humans). Hence, (Δ*pa*)^2^ is zero only within the bounds of statistical deviations. The classical approach to solve this problem within the ANOVA/MANOVA framework is a modification of the *F-*test. The error term is hereby enhanced from *e* ^2^ to (*e* ^2^ + (Δ*pa*)^2^), and the effect is tested through *h* ^2^/(  *e* ^2^ + (Δ*pa*)^2^) instead of (2). The obvious disadvantage is the requirement of a higher level of the effect (Δ*a*)^2^ which has to rise significantly above the “noise-”term (*e* ^2^ + (Δ*pa*)^2^) as compared to the pure noise level due to *e* ^2^.

So an attempt to test (*h* ^2^ − (Δ*pa*)^2^)/e^2^ seems more favorable. But this might lead to a negative variance estimate, and it is not clear what effective degrees of freedom would have to be assigned to such a variance estimate.

### 2.3. Derivation of the F-Ratio Test Statistics 

To overcome this situation, we propose a statistic estimating the influence of the population with the help of a resampling technique. This statistic is based on the decreasing sample-to-sample variation when a fixed term is present as compared to the influence of purely random effects. 

Following [6], we rely (a) upon the classical error propagation rule and (b) upon the variance's variance. The error propagation rule is given as [13]

(8) $$\begin{array}{c} \sigma^{2}\left(g\left(x\right)\right)={\left(\frac{\partial g}{\partial x}\right)}^{2}\sigma^{2}\left(x\right)+\text{h}.\text{o}.\text{t}., \end{array}$$

where *g* is a smooth function, *x* a random variable, and h.o.t denote higher order terms. As usual in error propagation considerations, this formula neglects correlational and higher order effects. We mention further that neglecting variations around absolute means the variance of an empirical variance estimate may be written as [14]

(9) $$\begin{array}{c} {\hat{\sigma }}^{2}\left(\sigma^{2}\right)=\frac{2{\hat{\sigma }}^{4}}{df}. \end{array}$$

We denote the variance with ${\hat{\sigma }}^{2}$ and the empirical variance estimate with *σ* ^2^. This conforms to (3). 

As our last step (c), we decompose ${\hat{\sigma }}^{2}\left(h^{2}\right)$, the variance of the effect term

(10) $$\begin{array}{c} {\hat{\sigma }}^{2}\left(h^{2}\right)={\hat{\sigma }}^{2}\left({\left(\Delta pa\right)}^{2}\right)+{\hat{\sigma }}^{2}\left({\left(\Delta e\right)}^{2}\right). \end{array}$$

We assumed here all error terms to be uncorrelated to the rest. Essential here is the fact that the fixed effect does not contribute to the variation of *h* ^2^ and accordingly does not enter into the variance ${\hat{\sigma }}^{2}\left(h^{2}\right)$. With (9), (8), and (7), we may write the variance of the *F-*value defined in (3) as

(11) $$\begin{array}{c} \sigma^{2}\left(F\right)=F^{2}\left[\frac{\sigma^{2}\left(h^{2}\right)}{h^{4}}+\frac{\sigma^{2}\left(e^{2}\right)}{e^{4}}\right]+\text{h}.\text{o}.\text{t}. \end{array}$$

Using (8), this turns into

(12) $$\begin{array}{c} \sigma^{2}\left(F\right)=4F^{2}\left[\frac{\nu }{2df_{k}}+\frac{1}{2df_{ek}}\right] \end{array},$$

where *df*_*k*_ denotes the degrees of freedom of the effect considered, *df*_*ek*_ the corresponding error degrees of freedom, and *ν* is the ratio

(13) $$\begin{array}{c} \nu =\frac{\left({\left(\Delta pa\right)}^{2}+{\left(\Delta e\right)}^{2}\right)}{h^{2}}. \end{array}$$

We note that in the case of a pure random effect, *ν* becomes 1 and significant deviations towards a lower value point to a nonnegligible fixed effect. Equation (12) obviously suggests using the statistic *σ* ^2^(*F*)/*F* ^2^ to test for *ν*< 1. According to (12), the expectation value of this statistic is—under the null hypothesis *ν* = 1—given by 1/2*df*_*k*_ + 1/2*df*_*ek*_. To gain an estimate for *σ* ^2^(*F*), one may randomly resample, *m* times, a subset encompassing an equal number of probands from the original sample and, each time, find the *F-*value corresponding to the particular subset. So the method becomes a variant of the so-called delete-d jackknife [15]. It has been shown that the following quantity estimates *σ* ^2^(*F*) up to a factor [16, 17]

(14) $$\begin{array}{c} \sigma^{2}\left(F\right)=\frac{1}{m-1}\sum {\left(F_{j}-\langle F\rangle \right)}^{2}, \end{array}$$

where $E\left(\sigma^{2}\left(F\right)\right)={\hat{\sigma }}^{2}\left(F\right)$.The number of random splittings conducted is denoted as *m*, the average 〈*F*〉 is defined as

(15) $$\begin{array}{c} \langle F\rangle =\frac{1}{m\sum F_{j}} \end{array},$$

and *F*_*j*_ denotes the found *F-*value obtained from the *j*th of the *m* runs. The above mentioned factor depends on #probands and selected #probands per random sample [15]. (We abbreviate here “number of” with the symbol #.) This is important, because *p*, the probability of a person to appear in a particular random sample, increases with the ratio #probands per random sample/#probands per sample. In case of a small sample size, this may impose an additional restriction of the variance *σ* ^2^(*F*) [6].

The cumulative distribution of the ratios *σ* ^2^(*F*)/〈*F*〉^2^ will hence depend on the parameters (*df*_*k*_, *df*_*ek*_, #random splittings, #probands, #probands per random sample). The #random splittings, *m*, hereby influences the cumulative distribution because higher values for *m* lead to a narrower deviation around ${\hat{\sigma }}^{2}$(*F*). A deviation from a random result may be found by estimating the probability that a ratio *σ* ^2^(*F*)/〈*F*〉^2^ is by chance as small or smaller than the experimentally found estimate. If this probability is too low, the null hypothesis is rejected. We will come back to this point in the following section. 

These ideas may be extended to the multivariate case [6]. We note that the error effects may again be assumed to be uncorrelated. Therefore the off-diagonal elements of E are random with an expectation value of zero. Furthermore, the trace of the matrix HE^−1^ remains unchanged when the basis is changed such that the eigenvectors build the new basis. Hence the diagonal terms of HE^−1^ are expected to represent, on the average, the individual *F-*values, and the trace is the sum over the individual *F*_*i*_'s. In case of a fixed effect with only two states (*s *= 1) and *n* random variables, this leads to a multivariate *F* with value 1/*n *∑_*i*=1_^*n*^ *F*_*i*_. To test the null hypothesis H_0_ of having random effects only, we may again use the independence of *σ* ^2^ (*F*_*i*_) and find testat0, our first test statistic,

(16) $$\begin{array}{c} \text{teststat}0=\frac{\sum \sigma^{2}\left(F_{i}\right)}{4{\left(\sum F_{i}\right)}^{2}} \end{array},$$

whose distribution is a function of (*df*_*k*_, *df*_*ek*_, *n*, #random splittings, #probands, #probands per random sample). If random effects for the treatment term exist, things become a bit more complicated. In that case, the contributions of the individual *σ* ^2^(*F*_*i*_) may be unequal, and—in extremis—the sum may be dominated by one single term. A way to account for this effect is to consider *df*_eff_, the effective degrees of freedom. The effective degrees of freedom are defined as *df*_eff_ = (∑*σ*_*i*_^2^)/(∑(*σ*_*i*_^2^ / *df*_*i*_)) (cf. [8], Chapter 8). This quantity is minimized if one term is clearly dominant and maximized when there are equal contributions. 

**Figure 1:**
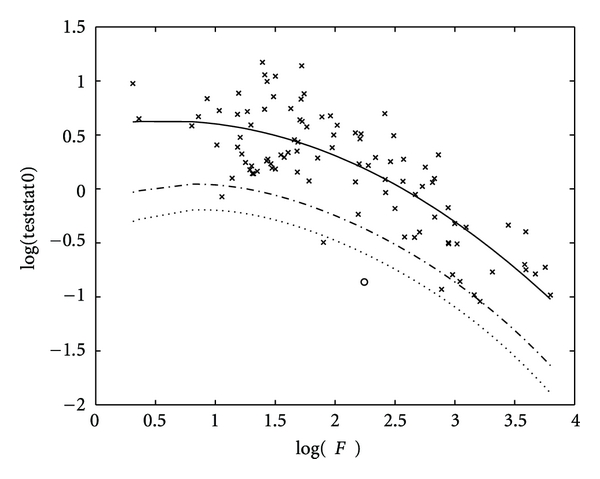
Outcome of an artificially generated signal with fixed effect (o) for our test statistics (testat0 (16) versus 〈*F*〉 (15), logarithmic scale) compared to outcomes of the corresponding random effects (*x*). The deviation from the expected value (solid line) of the latter is highly significant and below the 5% level (dash-dotted line) and even the 1% level (dotted line). The classical method according to Section 2.1 revealed the (insignificant) 13.95% level only. The proposed method recognizes the nonrandom effect correctly in this example while the classical approach does not.

As stated above, if an empirical value of teststat0 appears too low, one may conclude that there is a systematic nonrandom deviation in at least one variable between the treatment groups under consideration (see Figure 1). 

 In the case of a true multivariate statistic type, one has to replace the univariate individual *F-*values by the eigenvalues of HE^−1^ and modify testat0 into

(17) $$\begin{array}{c} \text{teststat}1=\frac{\sum_{i=1}^{s}\sigma^{2}\left(1/g_{i}\sum_{j=1}^{n}k_{i}^{j}F_{j}\right)}{4{\left(\sum_{i=1}^{s}1/g_{i}\sum_{j=1}^{n}k_{i}^{j}F_{j}\right)}^{2}} \end{array},$$

where *k*_*i*_^*j*^ *F*_*j*_ is the contribution of the individual univariate *F-*value *F*_*j*_ to the *i*th eigenvalue of (HE^−1^) adjusted with the degrees of freedom, namely, *c*_*i*_ *df*_*e*_/  df_*h*_. This statistic depends on (*df*_*h*_, *df*_*e*_, *n*, #simulations, #probands, #probands per random sample, stattype, *df*_eff_). If stattype, the statistics type, is Hotelling's statistics, this obviously becomes equivalent to the *s* = 1 case because *g*_*i*_ = const. and *F* = ∑_*i*=1_^*s*^ *c*_*i*_ *df*_*e*_/*df*_*h*_ (cf.  Section 2.1). In absence of a between-variable effect, one will have

(18) $$\begin{array}{c} \text{testat}1=\frac{\sigma^{2}\left(F_{\text{multi}}\right)}{4F_{\text{multi}}^{2}}. \end{array}$$

This suggests two normalized versions of our test statistic in the following way:

(19) $$\begin{array}{c} \text{teststat}1_{R}=\frac{\sum_{i=1}^{s}\sigma^{2}\left(1/g_{i}\sum_{j=1}^{n}k_{i}^{j}F_{j}\right)}{{4\left(\sum_{i=1}^{s}1/g_{i}\sum_{j=1}^{n}k_{i}^{j}F_{j}\right)}^{2}} \end{array}/\frac{\sigma^{2}\left(F_{\text{multi}}\right)}{4F_{\text{multi}}^{2}}.$$

The expectation value under the null hypothesis (i.e., having no multivariate effect) is 1, and the cumulative distribution depends on (*df*_*h*_, *df*_*e*_, *n*, #simulations, #probands, #probands per random sample, stattype). Significant deviations from 1 indicate that at least one variable shows a fixed effect or that a between-variable effect exists. 

As a last step, we extend (19) to an incremental test statistic. In the case of having already knowledge on certain measures displaying a multivariate effect, one may wish to test for the influence of an additional measure. We therefore modify the test statistic testat1_*R*_ into

(20) $$\begin{array}{c} \text{teststat}1_{M}=\frac{k^{2}\sigma^{2}\left(F_{c}\right)+\sigma^{2}\left(F_{\text{add}}\right)}{{4\left(kF_{c}+F_{\text{add}}\right)}^{2}}/\frac{\sigma^{2}\left(F_{\text{multi}}\right)}{4F_{\text{multi}}^{2}} \end{array},$$

where *k* is the number of those measures already showing a multivariate effect, and *F*_*c*_ is the *F-*value found with these measures. Our assumption of an existing effect implies *F*_*c*_> 1, because E(*F*_*c*_) > E(*F*_random_) and *σ* ^2^(*F*_*c*_) ≤*σ* ^2^(*F*_random_). Hence testat1_*M*_ tests the null hypothesis (*F*_*c*_> 1, *ν* = *ν*(*F*_c_)), that is, the additional variable has no influence. The cumulative distribution function then depends on (*df*_*h*_, *df*_*e*_, *n*, #simulations, #probands, #probands per random sample, *F*_*c*_, *σ* ^2^(*F*_*c*_), *df*_eff_, stattype) because E(*F*_c_) > E(*F*_random_) and *σ* ^2^(*F*_c_) ≤*σ* ^2^(*F*_random_). Because *σ* ^2^(*F*_*c*_) is assumed to be unequal to *σ* ^2^(*F*_add_), we must again consider the so-called effective degrees of freedom *df*_eff_ of the pooled variances.

The assumptions entering this incremental test are the same as in teststat1_*R*_. The null hypothesis states that the additional measure contributes its univariate *F*-value *F*_add_ to the trace while *F*_add_ is built up from nonfixed effects only. If the teststat1_*M*_ becomes unexpectedly high, this may be regarded as indicating an additional systematic effect due to the inclusion of this measure. If the statistic type is Hotelling's statistic, this becomes again equivalent to the *s* = 1 case. 

These statistics are useful answering questions like the following: “are there measures providing significantly to the treatment term?” and, if so, “which ones may be identified?” and “to what extent do they provide to the effect?” The knowledge of such measures and its contribution to the treatment effect allows one, for example, to select them and collect them with appropriate weights into a feature vector useable for discriminance or predictive purposes.

### 2.4. The Computational Scheme to Determine Confidence Intervals for the F-Ratio Test Statistics and Comparison with the Classical Approach 

The quantity of interest, namely, the distribution of the ratios *σ* ^2^(*F*)/*F* ^2^, must be evaluated numerically, and the dependence of the ratios from the number of random splittings and the number of persons involved calls for a calculation of the confidence intervals for each case. Generating the distribution of the *F*-ratios appropriately and, therefrom, the desired confidence interval is our method of choice to overcome this problem. This algorithm is basically a Monte Carlo technique generating *L* outcomes and their *F*-ratios. This leads to a population of *L* random deviates of the ratio *σ* ^2^(*F*)/〈*F*〉^2^ according to the appropriate null hypothesis (remember Figure 1). We note that both the *F*-value obtained for the whole sample as well as 〈*F*〉 (15) provide an estimate for *F* and calculating *σ* ^2^(*F*) and 〈*F*〉^2^ is done within the same procedure, so we prefer *σ* ^2^(*F*)/〈*F*〉^2^. From the population of the *L* ratios, one may derive a quantile and the associated probability *P*, for example, by building a histogram or ordering the population by rank and selecting the *P* · *L*th value. This value estimates the quantile above which *F*-ratios occur by chance with probability *P*.

#### 2.4.1. General Scheme

The general scheme of our algorithm is stated in more detail as follows [6].

1. Restate the model through a separation of the desired factor. The multivariate model describing our null hypotheses may be derived from (1) and may be formulated as
  (21) $$\begin{array}{c} {\text{Signal}}_{ijk}=\alpha_{i(j)}+\beta_{j}+{\text{error}}_{ijk}, \end{array}$$
  where Signal_*ij*_ denotes the (uni- or multivariate) measured quantities, *β*_*j*_ the random factor considered (e.g., different clinical groups), *α*_*i*_ and the other factor(s), which may implicitly depend on the random factor.
2. Determine/select the constants *k*, *L*, *m*, #*n*, *p*, stattype (if necessary) such that *L* is the number of deviates desired to estimate the quantile with acceptable accuracy, *m* is the number of random splittings needed for each deviate, #*n* the levels of the factor *β* (typically the number of persons involved, i.e., #probands), *p* the relative number of levels (or persons, i.e., #probands per random sample/#probands) entering one splitting, *k* the number of levels of *α*_*i*_, and stattype is again the multivariate statistic type. The values *k*, *m*, #*n*, *p*, stattype must conform to the setting with which the original data was analyzed.
3. Perform the Monte Carlo loop. This encompasses the following steps.
  - Generate a sequence of #*n* times *k* random numbers to mimic the random errors in (21). The amplitude must be chosen to match the value found for *e* ^2^ in the original analysis.
  - Generate another random #*n*-sequence to mimic the influence of the random factor. The amplitude must be chosen to match the null hypothesis. The random treatment effect assumed, (Δ*pa*)^2^, should be chosen such that 〈*F*〉 matches the found univariate outcome.
  - Add the different contributions to the simulated signal.
  - Build *m* random splittings and analyze it by the same procedures as the original sample was analyzed. Typically m is chosen to lie between 12 and 50. From the *m* splittings, build *σ* ^2^(*F*), 〈*F*〉^2^ (14), and (15), and the ratio *σ* ^2^(*F*)/〈*F*〉^2^. The analysis is normally done by means of a statistical software package estimating an appropriate *F*-value. This is sufficient for testat0. In the case of testat1, also build 〈*F*_multi_〉^2^, *σ* ^2^(*F*_multi_), and the ratios *σ* ^2^(*F*_multi_)〈*F*_multi_〉^2^ and (*σ* ^2^(*F*)/〈*F*〉^2^)/(*σ* ^2^(*F*_multi_)/〈*F*_multi_〉^2^). These are necessary for the different variants of testat1 (18)–(20).
  - Repeat steps (a) to (d) *L* times and gain therefrom empirically the quantile(s) of interest. As stated above, this may be done by means of a histogram or a rank ordered sequence obtained from the *L* *F-*ratios *σ* ^2^(*F*)/〈*F*〉^2^ and (*σ* ^2^(*F*)/〈*F*〉^2^)/(*σ* ^2^(*F*_multi_)/〈*F*_multi_〉^2^). Depending on the probability *P* associated with the quantile and the desired accuracy, *L* will typically be on the order of 10^2^,…,10^5^.

The statistic testat1_*M*_ (20) requires some attention with respect to (a) simulation and (b) effective degrees of freedom. This is because we estimate *σ* ^2^(*F*_*c*_), where *F*_*c*_ is expected to be larger than one due to the already recognized fixed or common effect and, therefore, *σ* ^2^(*F*_*c*_)<*σ*_random_^2^. 

**Figure 2:**
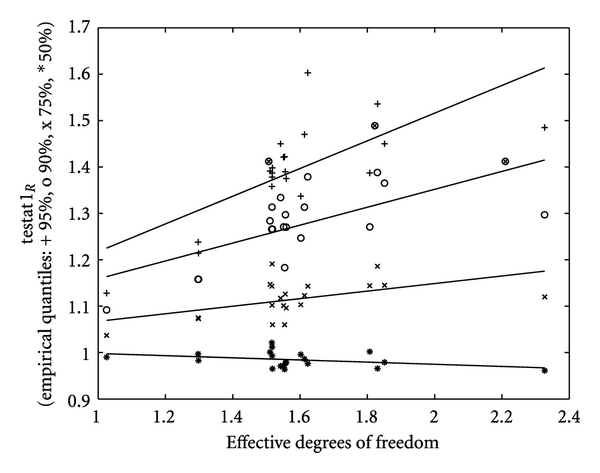
Variation of quantiles of the test statistics with the effective degrees of freedom *df*_eff_. 50% (∗); 75% (x); 90% (o); 95% (+) for a variety of simulations and their corresponding functional fit. The ⊗ denotes the results presented in Table 1. These are from left to right *χ*-*δ*, *χ* − *b*0, *χ*-*δ*-*α*.

*F*_*c*_ is carried over from the result obtained without the measure under consideration, so we test the additional measure under the constraints that the known effect equals *F*_*c*_ (or *F*_total_ = *F*_sample_total_). In the case that the measures contributing to *F*_*c*_ are expected to carry fixed effects, the model must also be adjusted with a fixed effect, such that the expected values *E*(*σ* ^2^(*F*)) and *E*(*σ* ^2^(*F*_*c*_)) match the corresponding values of the original sample. The quantiles must be derived at the point where *df*_eff_ matches *df*_eff_ of the original sample. This may be done by repeating step (e) thus collecting a population of empirical quantiles belonging to the same probability *P* and building a functional dependence quantile versus *df*_eff_ (cf.  Figure 2, where dependencies quantile_*P*_ = *a*_*P*_ +*b*_*P*_ · *df*_eff_ were fitted). The alternative is waiting until *L* results with approximately equal effective degrees of freedom emerged by chance. 

#### 2.4.2. Particular Settings 

The reconstruction of the model (21) is performed by generating streams of two types of uncorrelated random numbers from a normal distribution. The first type will mimic the error and has simulation parameters (0, *σ*_*e*_^2^), that is, the estimated squared mean of the error_*ij*_ of the original sample. The second type has simulation parameters (0, *σ*_*p*_^2^), that is, the average squared effect due to the probands. Both quantities may be read out from the output of the classical ANOVA/MANOVA analysis (cf.  Section 2.1) of the original sample. In this respect, the expected outcome of the simulation with the classical approach will correspond to the result obtained with the original sample, if the parameters *k* and #*n* also correspond to the original sample and the null hypothesis *H*_0_: “no presence of a fixed effect due to person group” is true. 

Our clinical sample consists of 30 persons from two clinical groups evaluated at four mental states ([18], see also Section 4.2). So we have *k* = 4 and #*n* = 30. Because the mental states have shown fixed effects in previous studies [18, 19], the simulated signals were offset by four fixed different levels. The amount of the offset values is not relevant, however, because the offset is fixed and the *F-*ratio test is set up to test for differences between the two groups. The offsets were introduced only to mimic better the original data. Hence a simulated person has four outcomes built by one choosing four times the same random deviate from (0, *σ*_*p*_^2^) plus four times a different random deviate from (0, *σ*_*e*_^2^) enriched with the state-specific offset. The first 15 simulated persons were labeled as group 1 and the last 15 labeled as group 2. The *F-*ratio tests were conducted with *m* = 30 and *p* = 2/3, if not stated otherwise. A Monte Carlo loop was normally evaluated with *L* = 100 for each stattype. Hence getting results for each of the stattypes testat0, testat1_*R*_, and testat1_*M*_ requires three different runs of the Monte Carlo loop. Roy's largest root (6) was used as the classical method, if not stated otherwise. 

**Figure 3:**
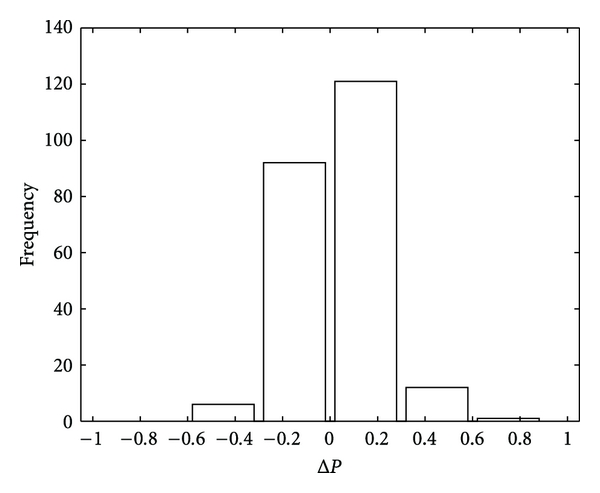
Comparison of the *F*-ratio test with the classical approach for 250 data samples. The probability of the spontaneous occurrence of the corresponding outcome is on the average smaller than with the classical approach. This is shown by the asymmetric distribution of Δ*P*, the differences between the two probabilities.

The *F*-ratio test statistic obviously requires more numerical efforts than the classical approach. So one could ask if its usage might be worth these efforts. We therefore tested the sensitivity of the *F*-ratio tests to the presence of fixed effects of person categories, that is, we tested for *H*_0_ in case when *H*_0_ is false. A comparison of runs on 250 different artificial data sets was made. We evaluated for each data set the probability that a test outcome as high or higher may occur by chance. This was done for both the classical test and the *F*-ratio test (applying a nonparametric method). Then we built for each set Δ*P* the difference between the probability according to the classical and the probability according to the *F*-ratio test. The resulting 250 values of Δ*P* were then sampled into a histogram. In case of equivalence of the two methods, one would expect a symmetric distribution around zero. Our data (Figure 3) show a significant deviation from a symmetric distribution towards the *F*-ratio test (*χ* ^2^ = 5.6, *P* = 0.02). The *F*-ratio test seems to be more sensitive to the presence of a fixed effect than the classical approach, thus a higher tendency to reject H_0_ in the case when the test should reject it. 

This seems not to be too surprising, however, because the deviations from the expected value of the quantity *σ* ^2^(*F*)/〈*F*〉^2^ occur in 4th power instead of the 2nd power as in the classical view. A further advantage of the *F*-ratio is its applicability to nonnormally distributed data because random number generation for nonnormal data bears no additional difficulties. 

Having established this as a method for an incremental inclusion of measures, we will now turn to the problem of using this knowledge to construct optimized feature vectors.

## 3. Hypothesis Weighting

Consider the outcomes of the tests above of, say, three measures which occur with different significance levels. We make the assumption that from these measures (or variables) the one with the least significance carries also the least information, while the others bear more information in accordance to their significance level. The problem with what weight they should enter into a feature vector is regarded from a Bayesian view. Bayes formula allows one to express a conditional probability *P*[*A*_*i*_ | *B*] with the conditional probabilities *P*[*B* | *A*_*j*_] through

(22) $$\begin{array}{c} P\left[A_{i}\;|\;B\right]=\frac{P\left[A_{i}\right]P\left[B\;|\;A_{i}\right]}{\sum_{j}P\left[A_{j}\right]P\left[B\;|\;A_{j}\right]} \end{array}.$$

This may be used to express the probability of a hypothesis H_*i*_ to be correct by means of the probabilities of the outcomes corresponding to the different hypotheses tested for. Consider two hypotheses *H*_0_ and *H*_1_ concerning the quality of the measures/variables. We would like to weight the hypotheses *H*_0_ (measures display no difference between groups) and *H*_1_ (measures display a difference between groups). The probability *P*(*H*_*i*_), namely, *H*_*i*_ being correct, appears as a natural weight for this hypothesis. Let *b* denote the empirical outcome of an *F*-ratio test as obtained with the Monte Carlo technique above. Let *B* denote the set of possible outcomes which deviate at least as much as the quantile belonging to the significance level *π*. If *b* exceeds this quantile it is also an element of *B*. The set *B* then allows for weighting hypotheses by means of (22). 

We may set the a priori probabilities *P*[*H*_0_] = 1 −*P*[*H*_1_] = *c* = 0.5, because we have no a priori preference neither for the hypothesis *H*_0_ nor an alternative *H*_1_. We may further assume the probability *P*[*B* | *H*_1_] = *c*_2_. The quantity *P*[*B* | *H*_0_]:= *π* is our present knowledge, namely, the probability assigned to find an outcome *b* within *B*, given *H*_0_, for example, *π* = 0.05, *π* = 0.1, and so forth. 

The probability of “*H*_0_ = true” given the set *B* may be written as  (22)

(23) $$\begin{array}{c} P\left[H_{0}\;|\;B\right]=\frac{c\pi }{c\pi +c_{2}\left(1-c\right)} \end{array}$$

and, similarly,

(24) $$\begin{array}{c} P\left[H_{1}\;|\;B\right]=\frac{c_{2}\left(1-c\right)}{c\pi +c_{2}\left(1-c\right)}. \end{array}$$

In general, we find the quantities *p*[*H*_1_^*i*^ | *B*] and may formally assign an “expected hypothesis” through the weighted mean

(25) $$\begin{array}{c} \bar{H}=\frac{\sum H_{1}^{i}p\left[H_{1}^{i}\;|\;B\right]}{\sum p\left[H_{1}^{i}\;|\;B\right]} \end{array}.$$

The formulation of an “expected alternative hypothesis” seems somewhat purely formal at this stage. However, if each hypothesis is intrinsically connected to a specific feature vector *f*_*i*_, this approach returns the expected feature vector $\bar{f}$ given the observation *B*, however,

(26) $$\begin{array}{c} \bar{f}=\frac{\sum f_{i}p\left[f_{i}\;|\;B\right]}{\sum p\left[f_{i}\;|\;B\right]}, \end{array}$$

because each feature vector *f*_*i*_ is spanned by its specific collection of measures

(27) $$\begin{array}{c} f_{i}={\left\{A,B,C,\ldots \right\}}_{i}. \end{array}$$

From the weights of the hypotheses one immediately also gets the weights of the measures. In the context of EEG time series analysis, the measures *A*, *B*, *C*,… denote quantities like correlation dimension, peak frequency, spectral band power, and so forth.

A simple weighting follows for the case of two possible alternative hypotheses. The likelihood ratio *P*[*H*_1_ | *B*]/*P*[*H*_0_ | *B*] then gives the weight with which the alternative is preferable to *H*_0_ when the weight of *H*_0_ is set to 1. It is expressed as

(28) $$\begin{array}{c} \frac{c_{2}\left(1-c\right)/\left(c\pi +c_{2}\left(1-c\right)\right)}{c\pi /\left(c\pi +c_{2}\left(1-c\right)\right)}=\frac{c_{2}\left(1-c\right)}{c\pi }. \end{array}$$

Now consider two alternatives *H*_1_^1^, H_1_^2^ and *P*[*B* ^1^ | *H*_0_] = *π*_1_, *P*[*B* ^2^ | *H*_0_] = *π*_2_, and *P*[*B* | *H*_1_^*i*^] = *c*_2_ for all *H*_1_^*i*^ (i.e., no preference for any alternative). Their likelihood ratio may be expressed through the ratio of their likelihood ratios against the null hypothesis [6]

(29) $$\begin{array}{c} \frac{c_{2}\left(1-c\right)/c\pi_{1}}{c_{2}\left(1-c\right)/c\pi_{2}}=\frac{\pi_{2}}{\pi_{1}}. \end{array}$$

This may be regarded as the weight with which the second alternative should enter when the weight of the first alternative is set to 1. If in addition *H*_1_^1^ is a subset of the *H*_1_^2^, that is, the variables assigned to *H*_1_^1^ are a subset of the variables assigned to *H*_1_^2^, this weighting applies to that part of *H*_1_^1^ which is not common to *H*_1_^2^. 

We have to note that the formulation of *c*_2_ is correct only when each probability *π*_*i*_ is small. If this is not the case, some correction might be required [6].

The application to the problem optimizing a feature vector is straightforward. The *i*th feature vector is regarded as the *i*th combination of measures corresponding to the *i*th hypotheses. To find the weights with which the variables enter the feature vector, we assume assigning the weight 1 to that combination of measures with the highest significance level. Taking into account the implicit dependence of *c*_2_ as stated above, the subsequent variables will enter with weights according to (26). If a probability (thus weight) falls close to zero, it may be set to zero which results in dropping that particular feature vector and its corresponding measures. This reduces the dimension of the optimal feature vector.

## 4. Application to the Problem Discriminating EEG States

### 4.1. Motivation of the Problem and Results of Earlier EEG Analysis

As an application, we choose the problem of distinguishing the EEG of the two proband groups taken from a neuropsychologically oriented study [19] by their EEG. This choice was motivated by the following: it is well known that schizophrenic patients show abnormalities compared to healthy controls when the so-called evoked potientials are studied [20–22]. This may point to a threshold regulation problem in the activation of the neural network in schizophrenics [23], and there might be differences in the metabolism of the frontal cortex [24, 25]. Therefore one may expect differences in the spontaneous EEG. Such differences were indeed reported repeatedly, for example, [26–28] using linear (FFT) or nonlinear (correlation dimension) analysis. 

An earlier study conducted with our proband samples (cf. below) revealed a significant difference between the two samples but only for a specific mental task [18]. While the EEG of the controls showed a drastic decrease in dimensionality, the EEG of the patients did not exhibit any pecularity. Other studies, however, pointed to the existence of a difference in the “eyes-closed quiet” state [2, 9]. The degree to which this difference is visible in the “eyes-closed quiet” state, that is, in absence of external activation, however, is not yet established and was examined with the method proposed here. 

### 4.2. Proband Sample and EEG Analysis

The neuropsychologically oriented EEG study consisted of two groups, namely, 15 acute hospitalized subjects diagnosed as schizophrenic and 15 controls in a healthy state. EEG measurements were repeated for four different mental tasks [19]. A trained clinical staff member ranked each patient's symptoms on a psychiatric rating scale, and the psychopharmaceuticals were noted. Both groups were exposed to the same mental tasks, while three 30-second segments of EEG were recorded [19]. We focus here mainly on the so-called “eyes-closed quiet" mental situation. The EEG were recorded according to the international 10–20 standard, which allows for the so-called parallel embedding scheme [2]. 

Our nonlinear EEG analysis follows a biparametric dimensional technique. In contrast to standard methods, this technique also considers attractor unfolding, and the outcomes provide several nonlinear measures, namely, the asymptotic correlation dimension (*b*_0_), the so-called unfolding dimension m*, and the relative unfolding (or Taken's) *χ* [9]. In addition, EEG analysis with conventional FFT techniques [29] was performed. This provided measures like *α*- or *δ*-power, that is, the spectral power from the so-called *α* ( 8–12 Hz) and *δ* (1–5 Hz) frequency band. A complete description of the proband samples, conditions, and technical settings is given elsewhere [18, 19]. With our experimental setup, the model consists of four fixed conditions (i.e., the four mental tasks) and two groups with 15 persons (i.e., patients and controls). According to our hypothesis, the influence of the group is in the focus of interest. Those persons building the two groups must be suspected to provide a sample-specific (or random) effect to the discriminant capacities between the groups (cf.  Section 2), however, and demand for the application of our scheme. In each group, 10 from the 15 persons where chosen for the simulation, that is, at the point *p* = 2/3.

### 4.3. Results

The findings listed in the Section 4.1 led us to hypothesize differences in the absence of stimulated activation or medication. Therefore we applied our method to the EEG outcomes to the “eyes-closed quiet” situation. The results obtained with the different test statistics of this setting are shown in Table 1. 

 From here one sees that the relative unfolding *χ* seems to play the role of a major indicator, because *χ* occurs in all combinations of Table 1. This result is in agreement with findings from an earlier study [2] and with previous results from our sample [18, 19]. The *δ* power seems to be the best spectral measure because it appears in two combinations. An effect on the *δ* band is also in agreement with older findings in the literature [30]. 

This let us expect a reliable discrimination between the two states, schizophrenic versus healthy, by means of the EEG outcomes, if a combination of measures is appropriately selected. Among the triple combinations, only *f*_*i*_ =(*χ*, *δ*-power, *α*-power) seems to carry information. The combination (*χ*, *δ*-power,  *b*_0_) did not show any remarkable effect. So the effect on *δ*-power and *b*_0_ seems somewhat opposite, and this combination was dropped. To discriminate between the two groups, it seems therefore reasonable to select the variables *χ*, *δ*-, and *α* power. The information obtained with these outcomes is used to build an appropriate feature vector. 

**Figure 4:**
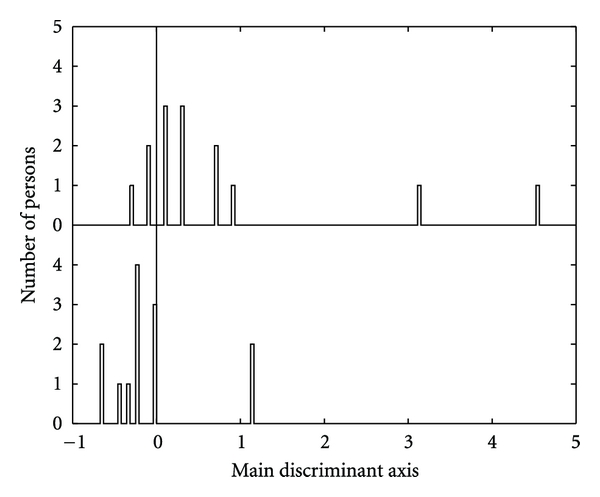
Discriminant analysis of EEG outcomes with weighted feature vector (eyes closed at rest). The number of persons is shown above the value on the main axis of the discriminant function where they appear. Upper: control group; lower: patient group (redisplayed from [6]).

Following Section 3 to find weights for feature vector components, we assume the 95% interval as significant and assign the weight 1. This conforms to *π*_1_ and *H*_1_^1^: *χ* and *δ*-power. Applying our considerations to the 90% solution (*π*_2_ = 0.1, *H*_1_^2^: *χ*, *δ*-power, *α*-power) reveals the weight 0.48. Hence, the variables *χ*, *δ* enter with weight 1.00 into the feature vector, while the variable *α* enters with weight 0.48 only. A discriminant analysis with this weighted feature vector reveals a correct classification with more than 81%. The result is displayed in Figure 4, where the outcome on the main axis of the discriminant function (essentially a rotation of the coordinate system [8], Chapter 18) is shown. The discriminant analysis could not be done on all 15 persons of each group. Due to failure to EEG-record quality requirements [19], one person of the control group and two persons of the patient group could not be evaluated, unfortunately. 

We note that our *F-*ratio test statistics with its ability to perform multivariate and incremental testing on fixed effects allowed for this weighting of feature vectors. Furthermore, we may regard this result as reliable because this variable weighting has been done based on the emergence of fixed effects, therefore not optimizing across random (or sample-specific) discriminant capacities. 

## 5. Discussion

We proposed and derived a computational scheme which is based on a random splitting method and which allows separating fixed and random effects in multivariate variance analysis. This approach seems to be advantageous in two respects. The classical method is implemented only for the univariate problem in most standard statistical software packages. So the decomposition of the effect matrix H into a fixed and a random effect requires additional matrix algebra programming efforts anyway. This may turn out to be a more difficult numerical problem than the generation of streams of random numbers. 

Secondly, the normality assumptions inherent to the classical test also remain true for the multivariate test, namely, normally distributed random deviations around the effect levels. If this is not true, the statistics to be used do not follow an *F-*distribution and may be unknown, thus preventing a classical significance test. 

In contrast, our method requires testing against quantiles derived from simulated outcomes. Thus the calculations can be done completely analogously when it seems more appropriate to use a distribution other than the normal distribution. Because our test statistic is based on relative ratios rather than absolute ratios, one might expect that an effect due to a particular distribution in the denominator will have a related effect in the numerator which could make our test statistic more robust. 

Our tests for partial inclusion followed a Bayesian weighting of hypothesis. This leads to an optimized feature vector. This feature vector comprises those measures relevant to the fixed effect being tested for. This exceeds the classical model selection because each measure enters with an appropriate weight between one and zero rather than in an all or none fashion. 

Another advantage of this approach is the simultaneous inclusion of linear and nonlinear measures. We note that the interpretation of the latter must be done with caution. It has been recognized for a long time that these measures are affected by noise and estimation errors when they are used for EEG analysis which then may circumvent their interpretation as chaos indicators (cf. e.g., [9, 31, 32] and the references concerning this matter therein). Despite this fact, these measures proved the ability to display individual properties of the EEG not seen with linear measures (cf. e.g., [2, 3]), and this is confirmed here. 

As was shown with our EEG data, the above mentioned properties of our methods allowed for a clear distinction (>81%) between the two proband groups, controls versus schizophrenic patients, in a resting state with eyes closed. Earlier results stating that *δ* and *χ* seem to differentiate between the two groups are confirmed, but such a clear result has not yet been found in previous studies.

## Figures and Tables

**Table 1:** Outcomes of *F-*ratio test statistics with a considerable significance level for EEG feature vectors.

Test statistic	Feature vector	*F*_multi_	Ratio	Test statistic	*df*_eff_	Significance
used	(measures)			value		level
teststat1_*R*_	*χ*, *δ*-power	6.168	0.233	1.412	1.507	>0.95
(19)						
teststat1_*R*_	*χ*, b0	10.393	0.145	1.489	1.822	>0.95
(19)						
teststat1_*R*_	*χ*, *δ*-power, *α*-power	6.890	0.158	1.416	2.21	>0.90
(19)						
teststat1_*M*_	*χ*, *δ*-power, *α*-power	6.890	0.158	1.192	2.21	⋍ 0.90
(20)						
